# Noncoding RNA Transcripts during Differentiation of Induced Pluripotent Stem Cells into Hepatocytes

**DOI:** 10.1155/2018/5692840

**Published:** 2018-08-19

**Authors:** Aniela Skrzypczyk, Stephanie Kehr, Ilona Krystel, Stephan H. Bernhart, Shibashish Giri, Augustinus Bader, Peter F. Stadler

**Affiliations:** ^1^Applied Stem Cell Biology and Cell Technology, Biomedical and Biotechnological Center, Leipzig University, Deutscher Platz 5, 04103 Leipzig, Germany; ^2^Bioinformatics Group, Department of Computer Science, and Interdisciplinary Centre for Bioinformatics, Leipzig University, Härtelstr. 1618, 04107 Leipzig, Germany; ^3^Transcriptome Bioinformatics Group at the Interdisciplinary Centre for Bioinformatics, Leipzig University, Härtelstr. 1618, 04107 Leipzig, Germany; ^4^Department of Plastic Surgery and Hand Surgery, University Hospital Rechts der Isar, Technische Universität München, Ismaninger Str. 22, 81675 Munich, Germany; ^5^Competence Center for Scalable Data Services and Solutions Dresden/Leipzig, German Centre for Integrative Biodiversity Research (iDiv), and Leipzig Research Center for Civilization Diseases, Universität Leipzig, Ritterstrasse 9-13, 04109 Leipzig, Germany; ^6^Max Planck Institute for Mathematics in the Sciences, Insel Strasse 22, 04103 Leipzig, Germany; ^7^Fraunhofer Institute for Cell Therapy and Immunology, Perlickstrasse 1, 04103 Leipzig, Germany; ^8^Department of Theoretical Chemistry, University of Vienna, Wahringerstrasse 17, 1090 Vienna, Austria; ^9^Center for RNA in Technology and Health, University of Copenhagen, Grønnegårdsvej 3, Frederiksberg C, Denmark; ^10^Santa Fe Institute, 1399 Hyde Park Road, Santa Fe NM 87501, USA

## Abstract

Recent advances in the stem cell field allow to obtain many human tissues in vitro. However, hepatic differentiation of induced pluripotent stem cells (iPSCs) still remains challenging. Hepatocyte-like cells (HLCs) obtained after differentiation resemble more fetal liver hepatocytes. MicroRNAs (miRNA) play an important role in the differentiation process. Here, we analysed noncoding RNA profiles from the last stages of differentiation and compare them to hepatocytes. Our results show that HLCs maintain an epithelial character and express miRNA which can block hepatocyte maturation by inhibiting the epithelial-mesenchymal transition (EMT). Additionally, we identified differentially expressed small nucleolar RNAs (snoRNAs) and discovered novel noncoding RNA (ncRNA) genes.

## 1. Introduction

Human iPSC technology provides a powerful tool for both regenerative medicine and development analysis. Stem cells hold the potential to recapitulate embryonic differentiation of many tissues in vitro. Moreover, differentiated cells can replace damaged or degenerated cells in vivo (reviewed by [1, 2]). The liver is a complex organ with a high variety of functions. It is essential for detoxification and bile production. End-stage liver diseases are associated with hepatocyte apoptosis [3]. Currently, there is no possible compensation for liver failure. For many patients, the only option to survive is through liver transplant, which is limited due to organ shortage. IPSCs could potentially be the source of cells for bioartificial liver devices or transplantations [4]. To avoid tumorigenesis and ensure proper function, iPSCs must be fully differentiated. A variety of hepatic differentiation protocols has been described [5, 6]. However, the process of hepatic differentiation still needs to be improved. After differentiation, cells express several mature hepatic markers and functions, but it has been shown that they resemble fetal hepatocytes [7]. miRNAs are well-known regulators of gene expression during liver development [8]. These 21-22-nucleotide-long molecules can affect expression of multiple genes simultaneously by binding to complementary regions of messenger RNAs (mRNA). This interaction causes degradation or repression of the target transcript. miR-122 is the most abundant miRNA in the liver, and it has been shown that overexpression of miR-122 can enhance hepatic maturation of fetal liver progenitors [9]. Another important group of noncoding RNAs (ncRNAs) is the snoRNAs. They act as guides for chemical modifications in other RNAs, mainly in ribosomal RNA (rRNA). Based on different sequence motifs and secondary structures, snoRNAs are divided into two types: CD box snoRNAs, guiding ribose methylation, and H/ACA box snoRNAs which guide pseudouridylation [10, 11]. Some specific snoRNAs are known to also act in a miRNA-like fashion [12–14]. In human tissues, snoRNAs have been observed to be subject to differential expression [15] and have recently attracted attention as biomarkers [16–18].

In this study, we explore the involvement of miRNAs and snoRNAs in the dynamics of hepatic differentiation to shed light on the molecular and regulatory mechanisms that underlie this complex process. We compare miRNA expression profiles of HLCs at two stages of differentiation with hepatocytes and discuss potential inhibitors of hepatic maturation. In addition, we identified novel ncRNAs in the transcriptome of the analysed cells.

## 2. Materials and Method

### 2.1. Cell Culture

Induced pluripotent stem cells (iPSCs) were obtained from foreskin fibroblast by reprogramming with episomal vectors containing genes OCT4, SOX2, NANOG, KLF4, L-MYC, Lin28, and shRNA-p53 and the miR-302/367 cluster, along with the GFP marker (System Biosciences). Cells were cultured at 37°C in 5% CO_2_ in Essential 8 Medium (Life Technologies). Detailed description of the protocol for generation and characterization of the cells is described in [19]. Obtained iPSCs were split using Versene (Life Technologies) and seeded into Geltrex-coated (Life Technologies) six-well plates to initiate hepatic differentiation.

### 2.2. Hepatic Differentiation

Hepatic differentiation was performed following the protocol described in [20]. Briefly, when cells reach 70% confluency, the medium was changed for RPMI1640 media containing B27 Supplements Minus Insulin (Invitrogen), 100 ng/mL Activin A (R&D Systems), 20 ng/mL fibroblast growth factor 2 (FGF2) (R&D Systems), and 10 ng/mL bone morphogenetic protein 4 (BMP4) (PeproTech) to induce endoderm. After 8 days of culture, dishes were moved to hypoxia (4% O_2_) in RPMI/B27 Supplement (Invitrogen) medium with 20 ng/mL BMP4 and 10 ng/mL FGF2 for 5 days. Next, the medium was changed to RPMI/B27 supplemented with 20 ng/mL hepatocyte growth factor (HGF, PeproTech) for an additional 5 days in hypoxia. The final stage of differentiation was in HCM hepatocyte culture medium (Lonza, but omitting the EGF) supplemented with 20 ng/mL Oncostatin M (R&D Systems) for 5 days in normoxia (21% O_2_). During that time, the medium was freshly prepared and changed daily.

### 2.3. Immunofluorescence

Cells were fixed for 15 min at room temperature (RT) in 4% paraformaldehyde solution Roti-Histofix (Carl Roth GmbH & Co. KG), then washed three times in PBS (Life Technologies), and blocked and permeabilized for 1 hour in PBS with 1% fetal bovine serum (Sigma-Aldrich) and 0.1% of saponin (Sigma-Aldrich). Cells were then incubated with primary antibodies overnight at 4°C, rinsed with PBS, and incubated with secondary antibodies for 1 hour at RT. DAPI was used as a nuclear counterstain (Thermo Scientific). Antibodies used for characterization were alpha-fetoprotein (Dako A0008, rabbit polyclonal), HNF4a (Abcam ab92378, rabbit monoclonal), albumin (R&D Systems mab1455, mouse monoclonal), and cytokeratin-18 (Abcam ab82254, mouse monoclonal). To validate the efficiency, cells were cultured on two-well slides (Thermo Fisher Scientific Inc.) and after hepatic differentiation stained as described above for HNF4a and ALB. Whole slides (four wells in total) were scanned. The image analysis tool ImageJ (Schneider et al., 2012) was used to measure the area of double-positive cells.

### 2.4. qPCR

Gene expression of hepatocyte-specific proteins (protein phosphatase 1 (PP1), human hepatocyte nuclear factor 4 (HNF4a), albumin (ALB), alpha-fetoprotein (AFP), alpha-1 antitrypsin (A1AT)) was validated using qPCR. The total RNA was isolated from cells at day 24 using RNeasy Mini Kit (Qiagen). RNA was reverse transcribed according to the manufacturer's protocol. Expression of the target mRNAs was quantified using Applied Biosystems 7500 Real-Time PCR System with SYBR Green PCR Master Mix. Each reaction was performed in triplicate under the following conditions: 95°C 5 min followed by 40 cycles of 95°C for 15 s denaturation, 60°C 15 s annealing, and 72°C for 30s extension. To choose endogenous control, the expression of 10 genes was compared. The primers were purchased from BIOMOL (HHK-1). The protein phosphatase 1 (PP1) gene was used as endogenous control as a gene with the smallest variance between samples. The Ct value was normalized against the endogenous control to obtain ∆Ct; we used the following formula for gene expression = 2^−∆Ct^, where ∆Ct = Ct (gene of interest average) − Ct (endogenous control average). The following primers were used: protein phosphatase 1 (PP1) F 5′-TTC ATC TGC ACT GCC AAG AC-3′, R 5′-TCG AGT TGT CCA CAG TCA GC-3; human hepatocyte nuclear factor 4 (HNF4a) F 5′-ATG GCT CTC CTG AGA GTG GA-3′, R 5′-CAG CGC AAG ACC TAA TGA CA-3′; albumin (ALB) F 5′-GAA ACA TTC ACC TTC CAT GC-3′, R 5′-ACA AAA GCT GCG AAA TCA TC-3′; alpha-fetoprotein (AFP) F 5′-CAT ATG TCC CTC CTG CAT TC-3′, R 5′-TTA AAC TCC CAA AGC AGC AC-3′; alpha-1 antitrypsin (A1AT) F 5′-ATG ATC TGA AGA GCG TCC TG-3′, R 5′-AGC TTC AGT CCC TTT CTC GT-3′; and PP1 F 5′-TTC ATC TGC ACT GCC AAG AC-3′, R 5′-TCG AGT TGT CCA CAG TCA GC-3′.

### 2.5. Periodic Acid-Schiff (PAS) Staining

Cells at day 24 of differentiation were stained using periodic acid-Schiff- (PAS-) staining system (Sigma-Aldrich) according to the manufacturer's instruction. Nuclei were counterstained with haematoxylin.

### 2.6. Indocyanine Green Uptake and Release

Indocyanine green (ICG, Cardiogreen, Sigma-Aldrich) was dissolved in DMSO (Sigma-Aldrich) and then added to the medium for 1 h. The final concentration of the resulting ICG solution was 1 mg/mL. After incubation, the medium was exchanged and images representing ICG uptake were taken. After 6 h, the functional ability of hepatocytes to remove the dye was inspected.

### 2.7. RNA Isolation and Sequencing

Total RNA, including short RNAs, was purified from frozen hepatocytes (pooled 10 donors HMCS10, GIBCO) and cells harvested at two different time points: day 20 of hepatic differentiation (hepatoblast stage of HD) and day 24, the last day of differentiation, using the miRNeasy Micro Kit (Qiagen) and quantified by NanoDrop spectrophotometer. Total RNA was used in the small RNA protocol with the TruSeq™ Small RNA sample prep kit v2 (Illumina) according to the instructions of the manufacturer. The barcoded libraries were size restricted between 140 and 165 bp, purified, and quantified using the Library Quantification Kit Illumina/Universal (KAPA Biosystems) according to the instructions of the manufacturer. Sequencing was performed with an Illumina HiScanSQ sequencer at the sequencing core facility of the IZKF Leipzig (Faculty of Medicine, Leipzig University).

### 2.8. Computational Analysis

The raw reads were prepared (quality control, adapter trimming) for mapping to the human genome assembly hg38 with segemehl [21], allowing multiple read mapping. Afterwards, the mapped reads were overlapped with the gene annotation (GENCODE v24) and the RepeatMasker track (retrieved from UCSC 2016/10/20) using rnacounter (J. Delafontaine, bbcf.epfl.ch) and bedtools [22], respectively. Additionally, up-to-date human snoRNA annotations were taken from literature [23]. The genomic regions that show expression signals but remain without annotations were aggregated to loci. Reads that map within a distance of 120 nucleotides were merged. Loci with a minimum coverage of 10 reads and a minimal length of 20 nucleotides were considered as putative novel ncRNAs.

### 2.9. Identification of Novel ncRNA Candidates

For loci with expression signals in all samples (day 20, day 24, and hepatocytes), we aimed to identify the type of transcript. First, we removed loci overlapping with nuclear insertions of mitochondrial sequences (NuMTs). The NuMT track available for the human hg19 assembly at UCSC Table Browser was mapped to hg38 using liftOver and intersected with the loci. Then we applied tRNAscan [24], snoReport [25], and RNAz 2.0 [26] to identify tRNAs, novel snoRNA candidates, and further putative ncRNAs. For each locus, we checked the conservation by searching for homologous sequences in other deuterostomian species using blast [27] (*E* value: 10^−3^1e 3, minimal base identity: 50%, minimal score: 60, and minimal length of query: 50%). Found homologous sequences were used as queries in the subsequent blast search in the next species. We rejected repetitive loci (having more than 100 accepted blast hits in a species) from further comparative analysis. Alignments containing all found homologous sequences were computed with MUSCLE [28]. Consensus secondary structures were computed using RNAalifold [29] under RALEE mode [30] in Emacs. To detect snoRNA sequences that have not been identified with snoReport, we first scanned the reads for putative box motifs using position weight matrices of the snoRNA boxes C, D, C′, D′, H, and ACA constructed from all annotated human snoRNAs. If a sequence harboured motifs C and D, or H and HACA in correct order and distance, we checked if the sequence is also able to fold into the typical snoRNA secondary structure using RNAfold. For sequences identified as putative snoRNAs in this manner, homologs were searched using the snoStrip pipeline [31].

### 2.10. Analysis of Differentially Expressed ncRNAs

Differentially expressed genes were identified using edgeR, a bioconductor software package [32] from replicated count data for every group pairwise comparison. Differentially expressed miRNAs and snoRNAs were selected by a false discovery rate (FDR) less than 0.001 and sorted by the adjusted fold change (including only log fold change higher than 2, |logFC| > 2).

### 2.11. Prediction of Target Genes and Pathways for Differentially Expressed miRNAs

In order to identify predicted targets of differentially expressed miRNAs, the DIANA mirPath tool V3.0 was used [33]. For every comparison, up to 50 significant miRNAs were analysed. DIANA-TarBase v7.0 was used to analyse gene interactions. Fisher's exact test was applied for statistical pathway union meta-analysis.

## 3. Results

### 3.1. Differentiation of iPSCs into Hepatocytes

**Figure 1:**
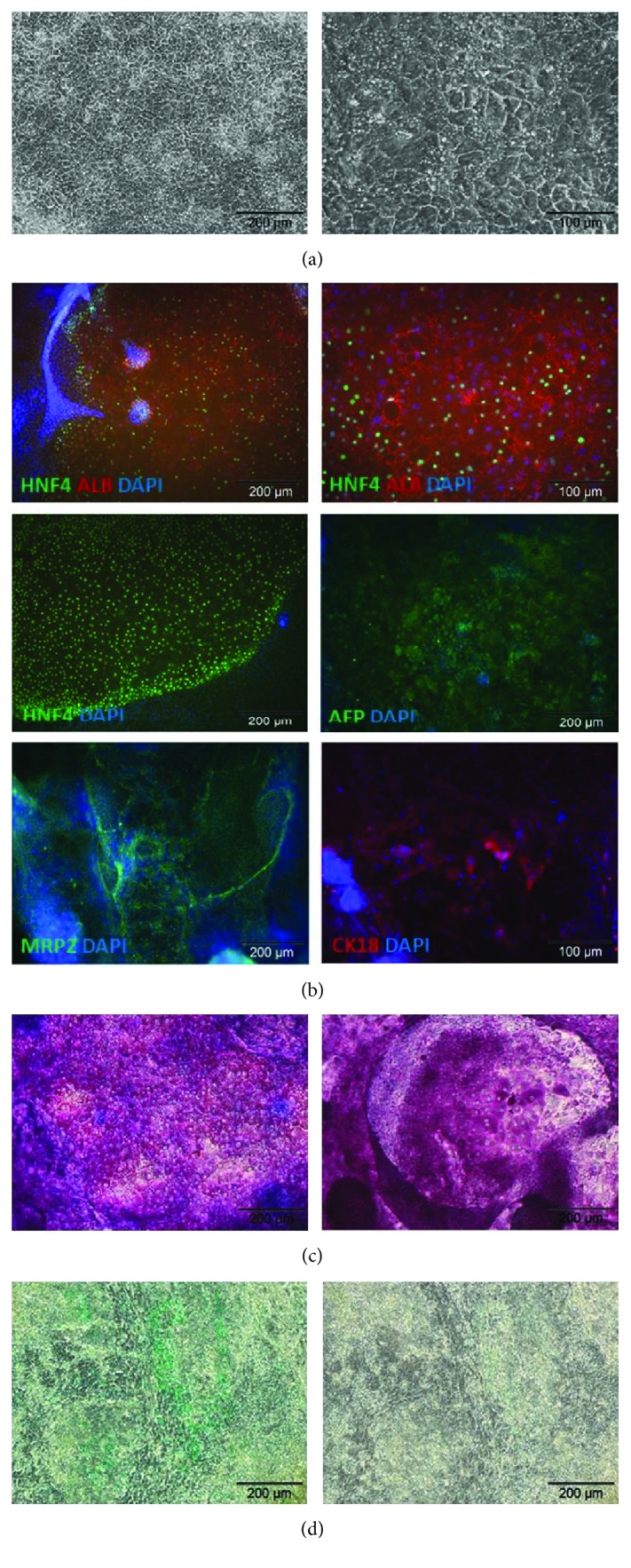
Characterization of hepatic-like cells (HLCs): (a) HLC morphology; (b) immunocytochemical detection of hepatic markers in HLCs HNF4a, ALB, AFP, MRP2, and CK18; (c) periodic acid-Schiff staining to detect glycogen storage in HLCs; (d) indocyanine green uptake and release in HLCs; green dye in cells indicate active take up of the dye and metabolism; representative images of three independent experiments.

**Figure 2:**
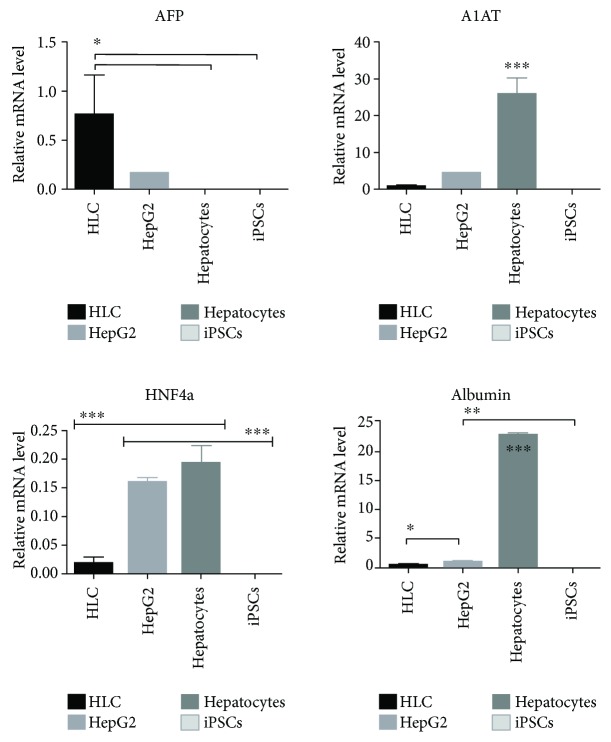
Quantitation of hepatic genes: AFP, A1AT, HNF4a, albumin mRNA levels by RT-qPCR analysis in HLCs, HepG2 (hepatocarcinoma cell line), hepatocytes, and iPSCs. The data shown originates from three separate experiments and are normalized to PP1 gene expression; statistical significant changes were calculated using one-way ANOVA with Tukey's multiple comparison test (^∗^ *p* < 0.05, ^∗∗^ *p* < 0.01, and ^∗∗∗^ *p* < 0.001 for significance).

**Figure 3:**
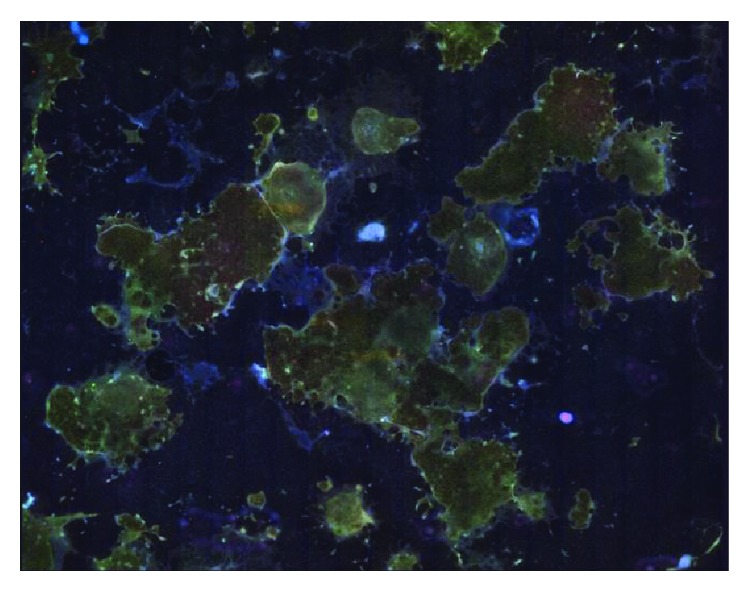
Whole slide scan after hepatic differentiation; cells stained for HNF4a (green), ALB (red), and nucleuses (DAPI); separate pictures with magnification ×400 were combined to create a virtual slide in order to calculate efficiency of the whole differentiation area; virtual slide of representative slide well.

During differentiation, stem cell morphology gradually changed towards the polygonal shape of hepatocytes. After 22 days of differentiation, we could observe binucleated cells and accumulation of lipid droplets (Figure 1(a)). The obtained HLCs exhibited a hepatic characteristic, including expression of the hepatic marker proteins albumin (ALB), hepatic nuclear factor 4 (HNF4), *α*-fetoprotein (AFP), multidrug resistance-associated protein 2 (MRP2), and cytokeratin-18 (CK18) (Figure 1(b)). Validation of HNF4, ALB, AFP, and alpha-1 antitrypsin (A1AT) with q-PCR resulted in clear expression signals (Figure 2). Further, the HLCs had the potential to store glycogen (PAS staining) (Figure 1(c)) and were also able to metabolise indocyanine green (ICG) (Figure 1(d)), both functions being specific to liver tissue, thus indicating successful differentiation. Efficiency of hepatic differentiation was evaluated using whole slide scanning. The area of cells double positive for HNF4 and ALB staining was measured using the image analysis tool ImageJ. We calculated that 30% of the total cell culture vessel was inhabited by cells positive for both hepatic markers (Figure 3).

### 3.2. RNA Analysis

**Figure 4:**
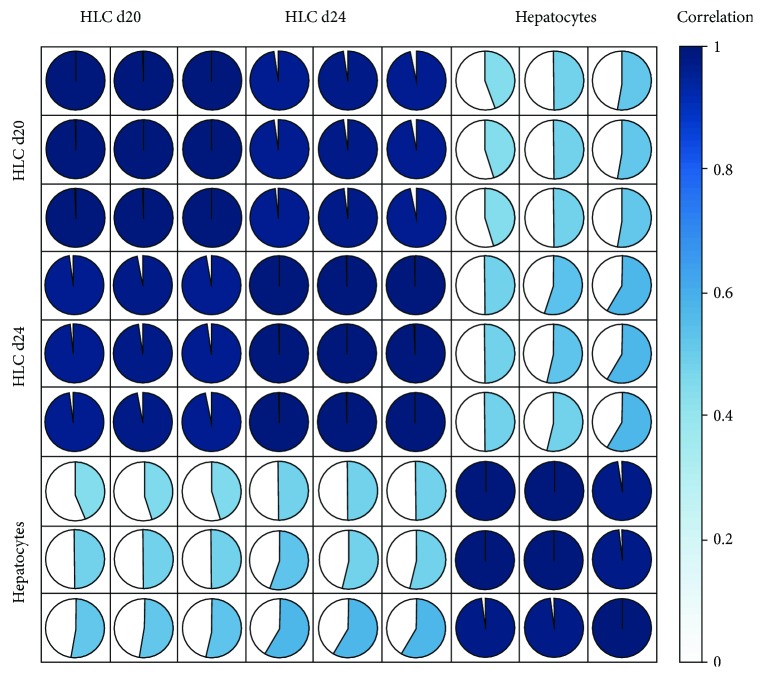
Correlation of gene expression levels between samples. Each pie chart represents the Pearson correlation (full pie chart and dark blue: correlation 1).

RNASeq of the different samples resulted in between 8.3 M and 25.2 M reads, of which 73% to 80% were longer than 17 nucleotides after adapter clipping. Between 92% and 94% of the clipped reads could be mapped (Supplementary Figure 1A). Between 345 k and 1.45 M reads were mapped to miRNAs, while between 4.14 M and 11.9 M reads were mapped to snoRNAs (Supplementary Figure 1B). Other types of transcripts were sequenced including rRNA (between 6.5% and 16.5%), snRNAs, lincRNAs (about 1%), and protein coding (between 0.6% and 7%) (Supplementary Figure 2). To visualize the consistency between replicates and global changes between the studied samples, hierarchical clustering of all detected ncRNAs was performed (Figure 4). This revealed a strong separation between hepatocytes and hepatic-like cells and good homogeneity within each group.

### 3.3. MicroRNAs during Differentiation of iPSC Cells

**Figure 5:**
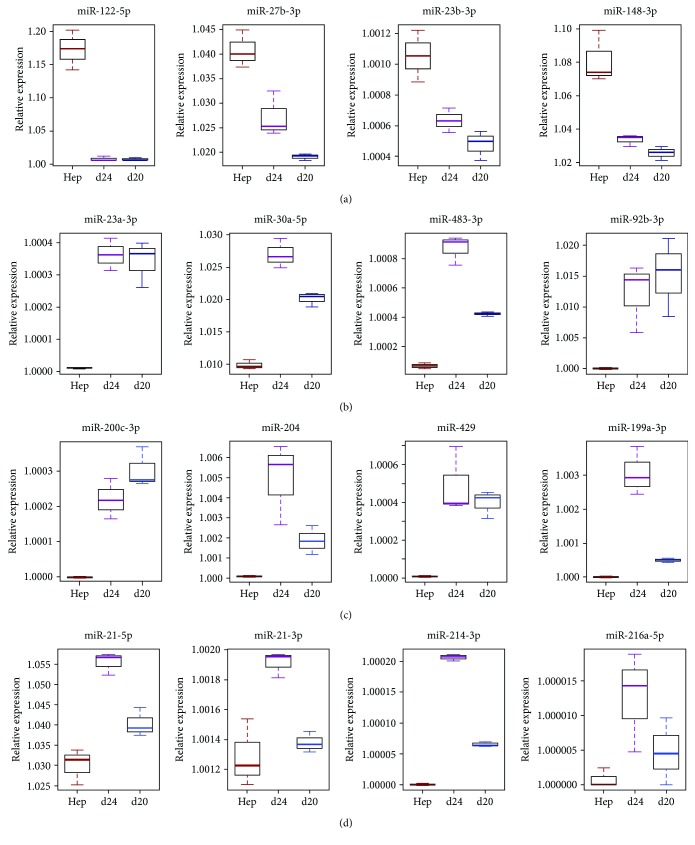
Detailed representation of expression changes of selected miRNAs in HLCs at days 20 and 24 of differentiation (d20 and d24, resp.) in comparison to hepatocytes: (a) hepatic-specific miRNAs (miR-122-5p, miR-27b-3p, miR-23b-3p, and miR-148-3p); miRNAs upregulated at day 24 of hepatic differentiation; (b) fetal liver-specific miRNAs (miR-23a-3p, miR-30a-5p, miR-483-3p, and miR-92b-3p); (c) miRNAs related to epithelial phenotype of HLCs (miR-200c-3p, miR-204, miR-429, and miR-199a-3p); (d) miRNAs connected to PI3K signaling (miR-21-5p, miR-21-3p, miR-214-3p, and miR-216a-5p).

We found about 20% (612/2812) of annotated miRNAs expressed (using a minimum of 10 reads as a cutoff) in at least one of the investigated samples. Hepatic-specific miR-122-5p, miR-27b-3p, miR-23b-3p, miR-148-3p, miR-146b-5p, and miR-194-5b were upregulated in hepatocytes. However, their expression in HLCs was decreased in comparison to hepatocytes (Figure 5). Nevertheless, elevated expression of mature hepatic miRNAs in HLC day 24 (d24) in comparison to HLC day 20 (d20) indicates hepatic lineage commitment during differentiation. The miRNAs upregulated in HLC day 24 in comparison to hepatocytes or HLC day 20 of differentiation have been reported to be specific for fetal hepatocytes: miR-23a-3p, miR-30a-5p, miR-483-3p, and miR-92b-3p. Upregulation of fetal liver miRNAs and expression of mature liver miRNAs in HLCs show that differentiated cells resemble a more fetal characteristic, which is in line with previous reports [7]. Remarkably, several miRNAs upregulated at the end of differentiation indicate an epithelial phenotype of HLCs. Those miRNAs which have previously been described as blocking epithelial to mesenchymal transition (EMT) were plotted separately: miR-200c-3p, miR-204-5p, miR-429, and miR-199a-3p. We also highlight miRNAs which have previously been shown to have increased expression levels during the last stage of hepatic differentiation of embryonic stem cells (ESCs) and are connected to PI3K signaling and differentiation: miR-21-3p, miR-21-5p, miR-214, and miR-216a [34].

**Figure 6:**
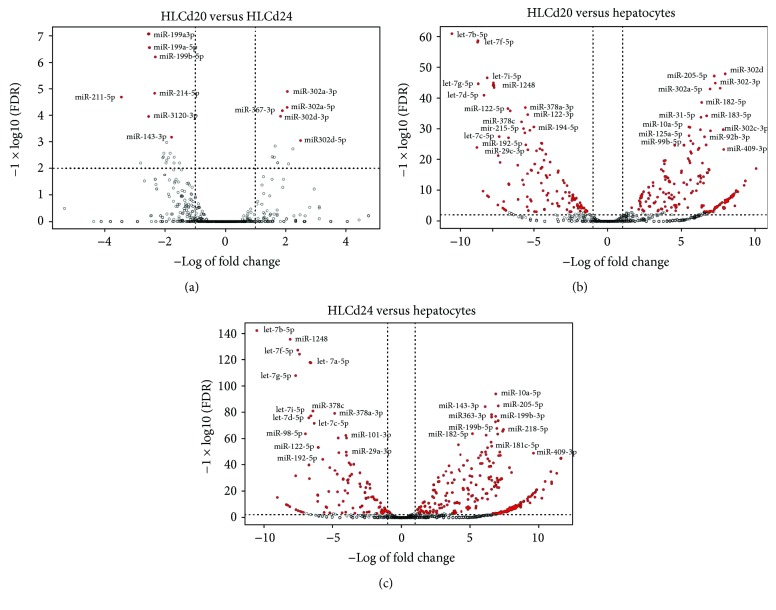
Volcano plots of miRNA expression in HLCs at day 20 and day 24 of differentiation and mature hepatocytes. The plots represent miRNA expression profiles of (a) HLCs at day 20 versus HLCs at day 24, (b) HLCs at day 20 versus hepatocytes, and (c) HLCs at day 24 versus hepatocytes; the *x*-axis indicates the difference of expression level on a log2 scale, while the *y*-axis represents corresponding adjusted *p* values (FDR) on a negative log10 scale; statistically significant differences extend vertically; red points indicate genes with significance level of FDR < 0.001; labels are given for the most significant differentially expressed miRNAs.

**Figure 7:**
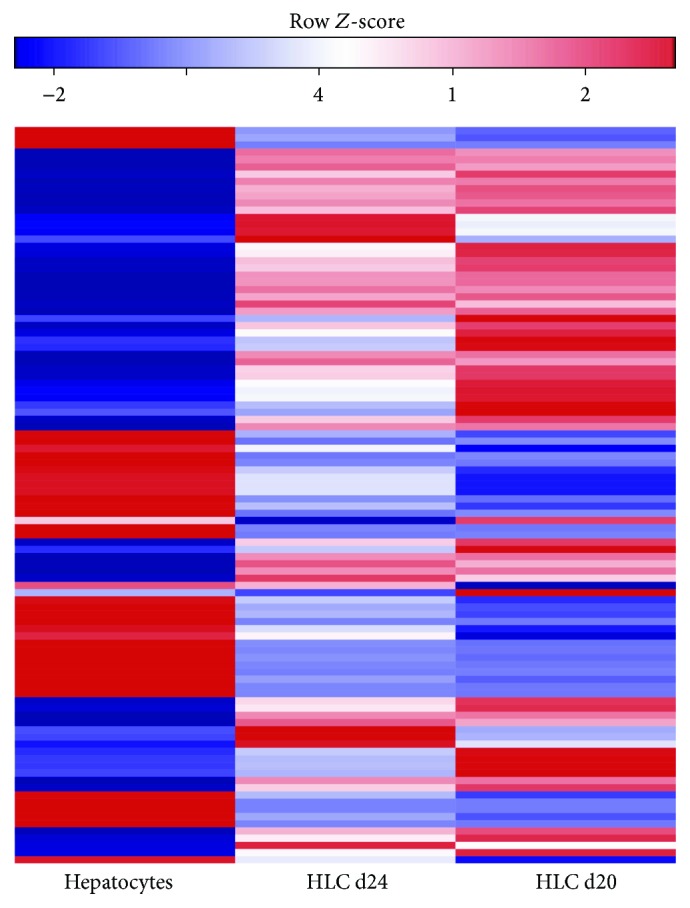
Heat map showing the differentially expressed miRNAs in shades of blue (low expression) and red (high expression) in HLCs at day 20 (d20) and day 24 (d24) of differentiation and hepatocytes (Hep).

We identify differentially expressed miRNAs between control hepatocytes and the different stages of differentiation (day 20 and day 24). Those with adjusted low *p* values (FDR) and at the same time high fold changes are marked and visualized in volcano plots (Figure 6). As expected, the miRNA expression changed during hepatic differentiation. In brief, 14 differentially expressed miRNAs were identified when HLCs were compared at day 20 and at day 24 of differentiation. Five miRNAs were downregulated in HLCs at day 24 including miR-367, miR-302, and miR-516. Another 19 miRNAs were upregulated, most remarkably miR-199a, miR-199b, miR-211, and miR-214. When mature hepatocytes were compared to HLCs at day 24, 228 miRNAs emerged as downregulated in the mature liver cells. This list contains in particular miR-181d, miR-199a, miR-214, miR-200c, and miR-205. Another 88 miRNAs were upregulated in hepatocytes: let-7b-5p, miR-29c, let-7f-5p, let-7g-5p, miR-612, and miR-195 among others. Three quarters of the differentially expressed miRNAs in hepatocytes compared with HLCs at day 24 were also identified as differentially expressed in hepatocytes compared to HLCs at day 20. To visualize the differentially expressed miRNAs, a heat map was prepared (Figure 7). A complete list of differentially expressed miRNAs is provided in Supplementary Table 1.

We analysed the enrichment of the KEGG gene ontology terms of miRNA target genes related to differentially expressed miRNAs. The resulting pathways are presented in Table 1. Target genes of upregulated miRNAs in HLCs are involved in prion diseases, fatty acid biosynthesis and metabolism, proteoglycans in cancer, ECM-receptor interaction, adherens junction, viral carcinogenesis, Hippo signaling pathway, transcriptional misregulation, and pathways in cancer. Hepatic upregulated miRNAs were found to regulate genes of pathways, which are typical for liver cells: hepatitis B, endodermal cell cancers, PI3K-Akt signaling pathway, focal adhesion, TGF-beta signaling pathway, and also genes of the thyroid hormone signaling pathway. Intriguingly, genes of the FoxO signaling pathway, protein processing in endoplasmic reticulum, and endocytosis were mostly targets of miRNAs differentially expressed between hepatocytes and HLCs at day 24 of differentiation.

### 3.4. snoRNAs during Differentiation of iPSC Cells

**Figure 8:**
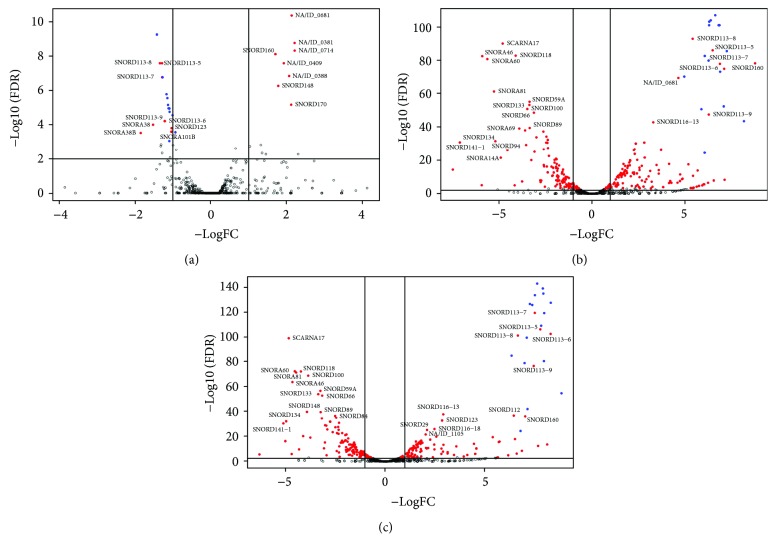
Volcano plots of snoRNA expression in HLC d20 and HLC d24 and hepatocytes: (a) HLCs at day 20 versus HLCs at day 24, (b) HLCs at day 20 versus hepatocytes, and (c) HLCs at day 24 versus hepatocytes. The *x*-axis indicates the difference of the expression level on a log2 scale, while the *y*-axis represents corresponding adjusted *p* values (FDR) on a negative log10 scale; statistically significant differences extend vertically; red points indicate genes with significance level of FDR < 0.001; blue points represent members of the SNORD114-family and are unlabelled. Labels are given for the remaining most significant differentially expressed snoRNAs.

**Figure 9:**
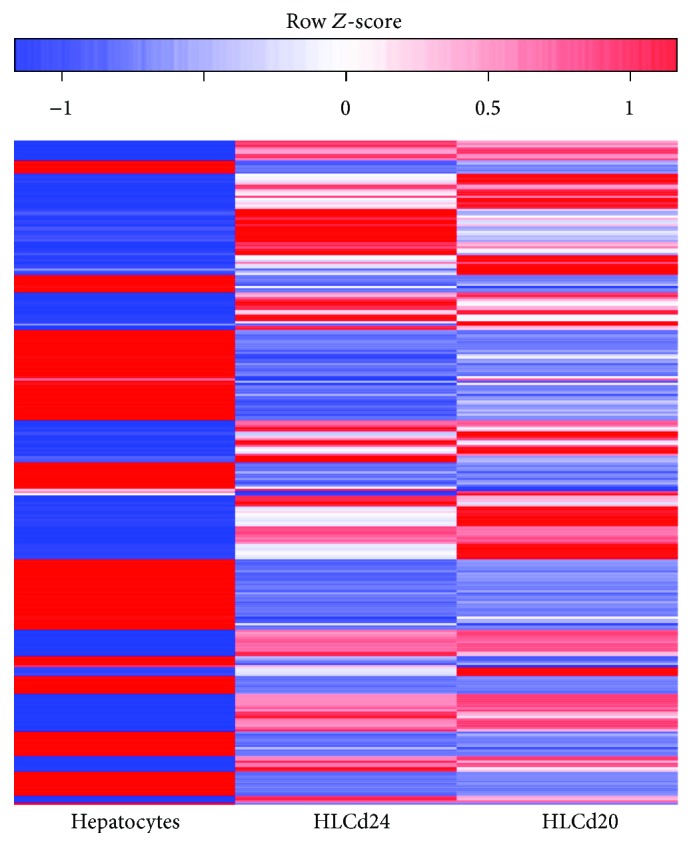
Heat map showing the differentially expressed snoRNAs in shades of blue (low expression) and red (high expression) in hepatocytes, HLC day 20 (HLCd20) and HLC day 24 (HLCd24).

We confirmed expression of 18 noncanonical SNORD-like (CD-box-like snoRNAs) and six candidate snoRNA genes reported in recent studies [23, 35]. These are expressed (minimum 10 reads) in at least one of the investigated samples. We identified many snoRNAs as differentially expressed snoRNAs during hepatocyte differentiation. Volcano plots representing these differentially expressed snoRNAs are shown in Figure 8. A total of 77.6% of the differentially expressed snoRNAs in hepatocytes compared with HLCs at day 20 are also found as differentially expressed in hepatocytes compared with HLCs at differentiation day 24. With the selected FDR cutoff of 0.001, 29 snoRNAs were differentially expressed between day 20 and day 24 of hepatic differentiation. Of those, 68% were canonical CD box snoRNAs, which corresponds to 44% of all canonical CD box snoRNAs. Another 19% are canonical H/ACA box snoRNAs, which corresponds to 30% of all canonical H/ACA box snoRNAs. The remaining 12% are noncanonical snoRNA transcripts, including, for example, CD-box-like and ALUACA snoRNAs. We visualized the differentially expressed snoRNAs as a heat map (Figure 9). A list of all differentially expressed snoRNAs is provided in Supplementary Table 1.

### 3.5. Novel ncRNA Predictions

In expressed loci that do not overlap gene annotations, we were able to identify 23 novel ncRNA candidates. Most of the newly predicted RNA sequences are conserved during evolution. One CD box snoRNA could only be identified in human and three snoRNA families of each type are identified as primate specific. Another seven predicted families are conserved also in other eutherian species. A list of all newly predicted ncRNA genes is provided in Table 2, and the conservation of novel genes is summarised in Supplementary Table 2.

### 3.6. snoRNA in Short Reads

**Figure 10:**
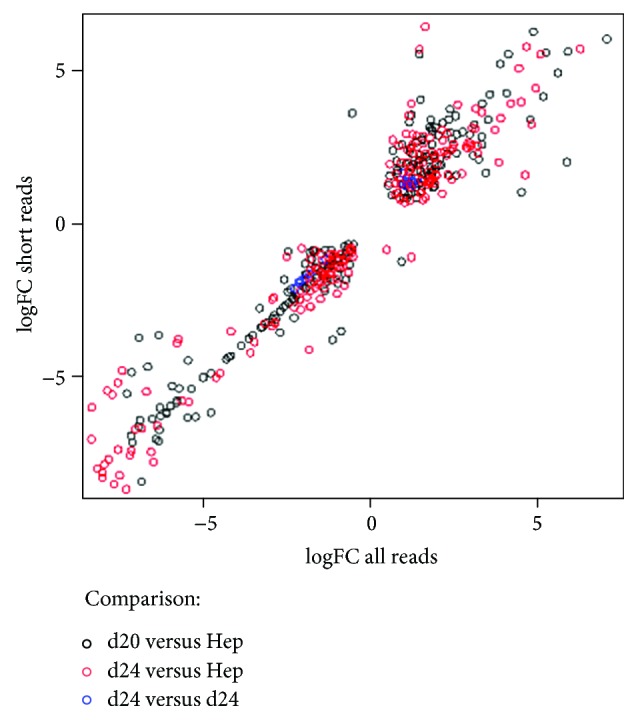
Correlation of fold changes between short reads and all reads mapping snoRNA genes; analysis of snoRNA genes with FDR below 0.001; *y*-axis—logarithmic transformation of fold change of short-read-only analysis, *x*-axis—logarithmic transformation of fold change when considering all reads; comparison of results from differential gene expression analysis of HLCs at d20 and hepatocytes (in black), HLCs at d24 and hepatocytes (in red), and HLCs at d20 and HLCs at d24 (in blue).

In order to investigate whether analysis of snoRNA short reads (≈20 nt + adapter) alone gives reasonable results for all snoRNA analysis (all snoRNA reads, full data set), we performed snoRNA differential expression analysis on short reads only. The results show that of differentially expressed snoRNAs from short reads (with an FDR of 0.001), 85 to 90% were also found differentially expressed in the full data set (containing about 4 times the number of snoRNA reads, mostly full length 50 nt). Of these still significantly different reads, only a maximum of 1% showed a different direction in change. This shows that miRNA sequencing, which usually gives snoRNA reads with adapters, can also be used to reliably investigate the differential expression of the snoRNAome (Figure 10).

## 4. Discussion

In this study, we analysed ncRNA profiles of iPSC-derived HLCs and compared them to profiles of hepatocytes to investigate potential inhibitors of hepatic maturation. The obtained HLCs express hepatic features; however, we could not attain high efficiency of differentiation. It was shown previously that hepatic differentiation efficiency varies between different protocols and depends on the used iPSC line [36]. The findings in this study are consistent with earlier data that HLCs differentiated from pluripotent stem cells have fetal characteristic [7].

We focused on comparison of miRNA profiles, which revealed that the gained HLCs undergo hepatic differentiation towards hepatic-like cells. Our results show upregulation of hepatic-specific miRNAs during the differentiation process in HLCs comparing day 20 with day 24. Additionally, the expression of fetal hepatic miRNAs was upregulated in HLCs especially on day 24 of differentiation when compared to hepatocytes. Analysis of differentially expressed miRNAs implicated that miRNAs whose expression is upregulated in HLCs are involved in differentiation, inhibition of proliferation, and maintenance of an epithelial phenotype.

Remarkably, analysis of differentially expressed miRNAs between HLCs at day 20 and day 24 showed that in HLCs at day 24, miR-199 is strongly upregulated, along with miR-214. Both miRNAs are regulators of skeleton formation, cardiogenesis, and cancer [37]. It has been shown that inhibition of miR-199a-5p improved hanging drop hepatic differentiation methods and liver repopulation ability of HLC derived from ESCs [38]. Furthermore, Möbus et al. also identified new target genes of miR-199a-5p, which are regulators of hepatocyte development. These findings might have important implications in the future when aiming to improve artificial hepatic maturation. miR-199a was also shown to be involved in liver fibrosis through deposition of extracellular matrix and profibrotic cytokine release, together with the miR-200 family [39, 40].

The miR-200 family (miR-200a, miR-200b, miR-200c, miR-141, and miR-429) is known as epithelial markers which were linked to inhibition of EMT by repressing ZEB1, ZEB2, and Snail [41]. Expression of those mRNAs is elevated in HLCs and can indicate that hepatic maturation and EMT is inhibited. During liver development, EMT is a natural process of hepatocyte differentiation, but it is also involved in carcinogenesis [42, 43]. Nevertheless, in a study of MSC-derived HLCs by Raut and Khanna (human umbilical cord Wharton's jelly-derived MSCs), it has been reported that EMT-related miRNAs are upregulated in the last days of hepatic differentiation [34]. Potential EMT inhibition during differentiation should be resolved in follow-up studies.

The HLCs obtained in this study had higher expression levels of miRNAs associated with phosphatidylinositol-3-kinase (PI3K) (miR-21, miR-214, and miR-216a) when compared to hepatocytes, which has also been described by Kim et al. [44]. Analysis with DIANA mirPath showed that upregulated miRNAs from hepatocytes also control the PI3K signaling pathway. This suggests that this pathway might be maintained by different miRNAs during hepatocyte differentiation and in the mature state.

miR-181 is another miRNA whose expression is highly upregulated in HLCs. It has been found highly abundant in fetal liver and has been linked to hepatocarcinoma [45]. In cancer cells, expression of epithelial cell adhesion molecule (EpCAM) was related to high miR-181 levels. This miRNA, however, targets epithelial gene caudal-type homeobox transcription factor 2 (CDX2) which promotes EMT. This suggests that expression of miR-181 might be essential in the balance between the epithelial and mesenchymal phenotypes in hepatocytes.

An analysis of KEGG pathways related to differentially expressed miRNAs in hepatocytes revealed that they control several pathways: the PI3K signaling pathway, as mentioned above, as well as focal adhesion, the TGF-beta signaling pathway, and the thyroid hormone signaling pathway. It was shown that transient hypothyroidism increases expression of miR-1, miR-206, miR-133a, and miR-133b in liver cells [46]. Interestingly, miR-1-3p and miR-133a were also identified in the group of differentially expressed miRNAs from HLCs. Differentially expressed, enriched miRNAs from HLCs compared to hepatocytes control fatty acid biosynthesis and metabolism, ECM-receptor interaction, proteoglycans in cancer, Hippo signaling pathway, adherens junction, lysine degradation, prion diseases, viral carcinogenesis, pathways in cancer, p53 signaling pathway, and cell cycle. The HLCs obtained in this study have fetal character, and tissue remodelling processes take place as a result of differentiation process. This result shows again that obtained HLCs are immature and undergoing many metabolic changes. Many of the differentially expressed miRNAs in HLCs are also involved in cancer. To clarify the miRNA interplay with genes and molecules, additional research is needed. The hepatic differentiation process is still limited. However, expression profiles obtained in this study will be helpful to understand the mechanism of differentiation and indicate the way of future research.

Strong evidence of differentially expressed snoRNAs was found in our dataset. A very useful methodological result in this context is that differential expression of snoRNAs can be detected and quantified reliably from miRNA-seq data and does not require sequencing of RNAs in a size range geared towards detecting snoRNAs.

Many of the differentially expressed snoRNAs belong to imprinted loci. Previously, hepatic snoRNAs from these regions were compared with ten other human tissues by [15]. All of those imprinted genes had low expression levels in the liver. Our results show that SNORD113, SNORD114, and SNORD116 are downregulated in hepatocytes comparing to HLCs, and members of SNORD115 are upregulated. This is in line with a study on the Prader-Willi syndrome locus where SNORD115 had higher expression levels than SNORD116 in the liver [47].

Finally, our data revealed 23 novel putative snoRNA families as well as four unclassified structured ncRNAs, most of which were evolutionarily young, suggesting that the repertoire of small structured RNAs is subject to rapid, lineage-specific expansions. For snoRNAs in particular, this points at functions beyond the ancient one as guide for chemical modification of ribosomal RNAs and snRNAs.

## Figures and Tables

**Table 1:** Gene ontology categories of differentially expressed miRNA targets (pathway union).

Comparison	KEGG pathway	*p* value	#genes	#miRNAs
Upregulated miRNAs in HLC d24 compared to HLC d20	Fatty acid biosynthesis	0	1	2
	ECM-receptor interaction	0	10	4
	Fatty acid metabolism	5.984709*e* – 10	2	2
	Proteoglycans in cancer	2.928118*e* − 09	31	3
	Hippo signaling pathway	0.0001180971	13	3
	Steroid biosynthesis	0.004048092	2	2
	Adherens junction	0.01782237	11	2
	Base excision repair	0.02708153	4	2

Upregulated miRNAs in HLC d20 compared to HLC d24	Lysine degradation	1.389225*e* − 08	9	2
	Chronic myeloid leukemia	0.0001602018	13	2
	Proteoglycans in cancer	0.0002270705	12	1
	Wnt signaling pathway	0.0006146658	12	1
	FoxO signaling pathway	0.003220974	18	2
	Cell cycle	0.003295535	9	1
	Pathways in cancer	0.004235095	17	1
	Progesterone-mediated oocyte maturation	0.03364343	8	1
	Oocyte meiosis	0.03398087	5	1
	Signaling pathways regulating pluripotency of stem cells	0.05899564	9	1

Upregulated miRNAs in HLC d20 compared to hepatocytes	Fatty acid biosynthesis	0	4	4
	ECM-receptor interaction	0	29	10
	Lysine degradation	0	26	17
	Proteoglycans in cancer	0	115	18
	MicroRNAs in cancer	2.065015*e* − 14	55	3
	Adherens junction	1.92849*e* − 10	56	14
	Fatty acid metabolism	2.435736*e* − 07	12	5
	Hippo signaling pathway	2.854731*e* − 07	76	15
	Prion diseases	3.567319*e* − 07	2	1
	Viral carcinogenesis	4.563306*e* − 07	90	10
	Pathways in cancer	7.078609*e* − 05	155	11
	Cell cycle	0.0002485214	62	9
	p53 signaling pathway	0.02762094	42	10
	Transcriptional misregulation in cancer	0.02815897	70	8

Upregulated miRNAs in hepatocytes compared to HLC d20	Fatty acid biosynthesis	0	4	7
	ECM-receptor interaction	0	39	15
	Lysine degradation	0	26	17
	Cell cycle	0	92	17
	Viral carcinogenesis	0	129	19
	Hippo signaling pathway	0	92	23
	Proteoglycans in cancer	0	140	23
	Pathways in cancer	1.110223*e* − 16	235	20
	Adherens junction	8.881784*e* – 16	59	20
	Hepatitis B	3.774758*e* − 15	83	13
	Chronic myeloid leukemia	3.940404*e* − 12	54	18
	Colorectal cancer	3.432521*e* − 11	43	17
	Glioma	9.944934*e* – 11	43	15
	Fatty acid metabolism	1.771966*e* – 06	14	8
	p53 signaling pathway	2.456315*e* – 06	47	16
	Small cell lung cancer	8.452771*e* – 06	57	14
	Oocyte meiosis	1.56543*e* − 05	65	12
	Thyroid hormone signaling pathway	2.996244*e* – 05	64	11
	Steroid biosynthesis	8.629469*e* – 05	7	12
	Prostate cancer	0.0004910115	65	13
	PI3K-Akt signaling pathway	0.001506451	118	10
	Focal adhesion	0.001709285	81	9
	TGF-beta signaling pathway	0.004683835	48	9

Upregulated miRNAs in HLC d24 compared to hepatocytes	Prion diseases	0	2	2
	Fatty acid biosynthesis	0	4	6
	Fatty acid metabolism	0	14	9
	Proteoglycans in cancer	0	107	15
	ECM-receptor interaction	0	34	18
	Adherens junction	2.136489*e* – 10	52	16
	Viral carcinogenesis	2.367215*e* – 07	84	11
	Hippo signaling pathway	8.686167*e* – 05	76	14
	Pathways in cancer	0.001098215	135	8
	Lysine degradation	0.001337938	24	10
	Transcriptional misregulation in cancer	0.003591186	53	7
	p53 signaling pathway	0.04591601	39	11

Upregulated miRNA hepatocytes compared to HLC d24	Fatty acid biosynthesis	0	4	7
	Hepatitis B	0	91	15
	ECM-receptor interaction	0	41	16
	Lysine degradation	0	27	18
	Cell cycle	0	93	18
	Viral carcinogenesis	0	132	18
	Pathways in cancer	0	247	21
	Hippo signaling pathway	0	91	22
	Proteoglycans in cancer	0	143	23
	Adherens junction	5.662137*e* – 15	58	19
	Chronic myeloid leukemia	1.465494*e* – 14	58	19
	Glioma	8.104628*e* – 14	46	16
	Colorectal cancer	8.277157*e* – 12	45	18
	p53 signaling pathway	2.698853*e* – 08	47	17
	Oocyte meiosis	1.888721*e* – 07	67	13
	Small cell lung cancer	5.78931*e* − 07	61	15
	Thyroid hormone signaling pathway	1.055741*e* – 05	64	11
	Prion diseases	2.777397*e* – 05	6	2
	Steroid biosynthesis	4.649816*e* – 05	10	11
	TGF-beta signaling pathway	6.111763*e* – 05	52	11
	Prostate cancer	0.0001105553	66	13
	Fatty acid metabolism	0.0001381028	13	7
	PI3K-Akt signaling pathway	0.0001991286	138	11
	FoxO signaling pathway	0.001107838	84	15
	Focal adhesion	0.001779401	90	9
	Bladder cancer	0.002833066	27	12
	Melanoma	0.01182136	41	10
	Protein processing in endoplasmic reticulum	0.01436954	105	12
	Endocytosis	0.01445943	97	12

**Table 2:** List of novel ncRNA gene candidates.

Position	Type	Host gene
chr1:113824553-113824673	HACA-snoRNA	PTPN22
chr1:181362152-181362263	HACA-snoRNA	—
chr1:40773163-40773278	HACA-snoRNA	—
chr1:153969534-153969592	CD-snoRNA	CREB3L4
chr3:168093129-168093244	HACA-snoRNA	GOLIM4
chr3:79560919-79561042	HACA-snoRNA	ROBO1
chr5:163294865-163294994	HACA-snoRNA	RP11-541P9.3
chr5:6757562-6757670	HACA-snoRNA	—
chr7:33591095-33591209	HACA-snoRNA	BBS9
chr9:122744852-122744927	CD-snoRNA	—
chr11:71300629-71300727	CD-snoRNA	—
chr11:98956624-98956737	CD-snoRNA	—
chr13:59018873-59018998	HACA-snoRNA	—
chr17:39725613-39725692	CD-snoRNA	ERBB2
chr21:39475295-39475353	CD-snoRNA	SH3BGR
chrY:6441667-6441790	HACA-snoRNA	—
chr5:116653307-116653431	CD-snoRNA	—
chr9:79487404-79487526	CD-snoRNA	—
chr10:30457496-30457617	CD-snoRNA	MAP3K8
chr11:2224892-2225019	RNAz	—
chr16:636684-636856	RNAz	MCRIP2
chr5:97539290-97539410	RNAz	LINC01340
chr8:27942407-27942528	RNAz	SCARA5

## Data Availability

The data used to support the findings of this study are available from the corresponding author upon request.

## Supplementary Materials

Supplementary Materials Figure 1: statistics of RNASeq. (A) Total number of sequenced reads, reads after clipping, and mapped reads; both symbolised all reads obtained after sequencing. The bar showing clipped reads contains reads that could be processed by cutting the helper sequences. Mapped read bar shows reads aligned to a reference genome. Clipped and mapped reads are divided into reads containing an adapter (length below 50 nt) and reads that do not contain an adapter (length ≥ 50 nt). (B) Percentage of mapped reads with an adapter; mapped reads of miRNA and snoRNA are divided into reads containing an adapter (length below 50 nt) and reads that do not contain an adapter (length ≥ 50 nt); HLCd20 and HLCd24 show reads from HLC day 20 and HLC day 24, respectively, of differentiation, in comparison to reads from hepatocytes. Figure 2: percentage of different transcript types in the sequencing; identified ncRNA without miRNA and snoRNA transcripts which were successfully mapped and overlapped genome annotations. HLCd20 and HLCd24 show transcripts from HLC day 20 and HLC day 24, respectively, of differentiation, in comparison to transcripts from hepatocytes. Table 1: list of differentially expressed miRNA and snoRNA. Table 2: conservation of snoRNA candidates.

## References

[1] Kimbrel E. A., Lanza R. (2015). Current status of pluripotent stem cells: moving the first therapies to the clinic. *Nature Reviews Drug Discovery*.

[2] Kamao H., Mandai M., Okamoto S. (2014). Characterization of human induced pluripotent stem cell-derived retinal pigment epithelium cell sheets aiming for clinical application. *Stem Cell Reports*.

[3] Malhi H., Gores G. J. (2008). Cellular and molecular mechanisms of liver injury. *Gastroenterology*.

[4] Tsolaki E., Yannaki E. (2015). Stem cell-based regenerative opportunities for the liver: state of the art and beyond. *World Journal of Gastroenterology*.

[5] Sgodda M., Mobus S., Hoepfner J. (2013). Improved hepatic differentiation strategies for human induced pluripotent stem cells. *Current Molecular Medicine*.

[6] Szkolnicka D., Hay D. C. (2016). Concise review: advances in generating hepatocytes from pluripotent stem cells for translational medicine. *Stem Cells*.

[7] Baxter M., Withey S., Harrison S. (2015). Phenotypic and functional analyses show stem cell-derived hepatocyte-like cells better mimic fetal rather than adult hepatocytes. *Journal of Hepatology*.

[8] Liu D., Fan J., Zeng W., Zhou Y., Ingvarsson S., Chen H. (2010). Quantitative analysis of miRNA expression in several developmental stages of human livers. *Hepatology Research*.

[9] Doddapaneni R., Chawla Y. K., Das A., Kalra J. K., Ghosh S., Chakraborti A. (2013). Overexpression of microRNA-122 enhances in vitro hepatic differentiation of fetal liver-derived stem/progenitor cells. *Journal of Cellular Biochemistry*.

[10] Dupuis-Sandoval F., Poirier M., Scott M. S. (2015). The emerging landscape of small nucleolar RNAs in cell biology. *Wiley Interdisciplinary Reviews: RNA*.

[11] Kiss T. (2002). Small nucleolar RNAs: an abundant group of noncoding RNAs with diverse cellular functions. *Cell*.

[12] Ender C., Krek A., Friedländer M. R. (2008). A human snoRNA with microRNA-like functions. *Molecular Cell*.

[13] Brameier M., Herwig A., Reinhardt R., Walter L., Gruber J. (2011). Human box C/D snoRNAs with miRNA like functions: expanding the range of regulatory RNAs. *Nucleic Acids Research*.

[14] Burroughs A. M., Ando Y., de Hoon M. L. (2011). Deep-sequencing of human Argonaute-associated small RNAs provides insight into miRNA sorting and reveals Argonaute association with RNA fragments of diverse origin. *RNA Biology*.

[15] Castle J. C., Armour C. D., Löwer M. (2010). Digital genome-wide ncRNA expression, including SnoRNAs, across 11 human tissues using polyA-neutral amplification. *PLoS One*.

[16] Ho L., Lange G., Zhao W. (2014). Select small nucleolar RNAs in blood components as novel biomarkers for improved identification of comorbid traumatic brain injury and post-traumatic stress disorder in veterans of the conflicts in Afghanistan and Iraq. *American Journal of Neurodegenerative Disease*.

[17] Su J., Liao J., Gao L. (2016). Analysis of small nucleolar RNAs in sputum for lung cancer diagnosis. *Oncotarget*.

[18] Steinbusch M. M. F., Fang Y., Milner P. I. (2017). Serum snoRNAs as biomarkers for joint ageing and post traumatic osteoarthritis. *Scientific Reports*.

[19] Skrzypczyk A., Giri S., Bader A. (2016). Generation of induced pluripotent stem cell line from foreskin fibroblasts. *Stem Cell Research*.

[20] Yu Y., Liu H., Ikeda Y. (2012). Hepatocyte-like cells differentiated from human induced pluripotent stem cells: relevance to cellular therapies. *Stem Cell Research*.

[21] Hoffmann S., Otto C., Kurtz S. (2009). Fast mapping of short sequences with mismatches, insertions and deletions using index structures. *PLoS Computational Biology*.

[22] Quinlan A. R. (2014). BEDTools: the Swiss-army tool for genome feature analysis. *Current Protocols in Bioinformatics*.

[23] Jorjani H., Kehr S., Jedlinski D. J. (2016). An updated human snoRNAome. *Nucleic Acids Research*.

[24] Schattner P., Brooks A. N., Lowe T. M. (2005). The tRNAscan-SE, snoscan and snoGPS web servers for the detection of tRNAs and snoRNAs. *Nucleic Acids Research*.

[25] Hertel J., Hofacker I. L., Stadler P. F. (2008). SnoReport: computational identification of snoRNAs with unknown targets. *Bioinformatics*.

[26] Gruber A. R., Findeiß S., Washietl S., Hofacker I. L., Stadler P. F. (2010). RNAz 2.0: improved noncoding RNA detection. *Pacific Symposium on Biocomputing*.

[27] Altschul S. F., Gish W., Miller W., Myers E. W., Lipman D. J. (1990). Basic local alignment search tool. *Journal of Molecular Biology*.

[28] Edgar R. C. (2004). MUSCLE: multiple sequence alignment with high accuracy and high throughput. *Nucleic Acids Research*.

[29] Bernhart S. H., Hofacker I. L., Will S., Gruber A. R., Stadler P. F. (2008). RNAalifold: improved consensus structure prediction for RNA alignments. *BMC Bioinformatics*.

[30] Griffiths-Jones S. (2005). RALEE—RNA alignment editor in Emacs. *Bioinformatics*.

[31] Bartschat S., Kehr S., Tafer H., Stadler P. F., Hertel J. (2013). snoStrip: a snoRNA annotation pipeline. *Bioinformatics*.

[32] Robinson M. D., McCarthy D. J., Smyth G. K. (2010). edgeR: a bioconductor package for differential expression analysis of digital gene expression data. *Bioinformatics*.

[33] Vlachos I. S., Zagganas K., Paraskevopoulou M. D. (2015). DIANA-miRPath v3.0: deciphering microRNA function with experimental support. *Nucleic Acids Research*.

[34] Raut A., Khanna A. (2017). High-throughput sequencing to identify microRNA signatures during hepatic differentiation of human umbilical cord Wharton’s jelly-derived mesenchymal stem cells. *Hepatology Research*.

[35] Kishore S., Gruber A. R., Jedlinski D. J., Syed A. P., Jorjani H., Zavolan M. (2013). Insights into snoRNA biogenesis and processing from PAR-CLIP of snoRNA core proteins and small RNA sequencing. *Genome Biology*.

[36] Kajiwara M., Aoi T., Okita K. (2012). Donor-dependent variations in hepatic differentiation from human-induced pluripotent stem cells. *Proceedings of the National Academy of Sciences of the United States of America*.

[37] Gu S., Chan W.-Y. (2012). Flexible and versatile as a chameleon—sophisticated functions of microRNA-199a. *International Journal of Molecular Sciences*.

[38] Möbus S., Yang D., Yuan Q. (2015). MicroRNA-199a-5p inhibition enhances the liver repopulation ability of human embryonic stem cell-derived hepatic cells. *Journal of Hepatology*.

[39] Murakami Y., Toyoda H., Tanaka M. (2011). The progression of liver fibrosis is related with overexpression of the miR-199 and 200 families. *PLoS One*.

[40] Jiang X.-P., Ai W. B., Wan L. Y., Zhang Y. Q., Wu J. F. (2017). The roles of microRNA families in hepatic fibrosis. *Cell & Bioscience*.

[41] Gregory P. A., Bert A. G., Paterson E. L. (2008). The miR-200 family and miR-205 regulate epithelial to mesenchymal transition by targeting ZEB1 and SIP1. *Nature Cell Biology*.

[42] Yoshida G. J. (2016). Emerging role of epithelial-mesenchymal transition in hepatic cancer. *Journal of Experimental & Clinical Cancer Research*.

[43] Du R., Wu S., Lv X., Fang H., Wu S., Kang J. (2014). Overexpression of brachyury contributes to tumor metastasis by inducing epithelial-mesenchymal transition in hepatocellular carcinoma. *Journal of Experimental & Clinical Cancer Research*.

[44] Kim N., Kim H., Jung I., Kim Y., Kim D., Han Y. M. (2011). Expression profiles of miRNAs in human embryonic stem cells during hepatocyte differentiation. *Hepatology Research*.

[45] Ji J., Yamashita T., Budhu A. (2009). Identification of microRNA-181 by genome-wide screening as a critical player in EpCAM–positive hepatic cancer stem cells. *Hepatology*.

[46] Dong H., Paquette M., Williams A., Zoeller R. T., Wade M., Yauk C. (2010). Thyroid hormone may regulate mRNA abundance in liver by acting on microRNAs. *PLoS One*.

[47] Galiveti C. R., Raabe C. A., Konthur Z., Rozhdestvensky T. S. (2015). Differential regulation of non-protein coding RNAs from Prader-Willi syndrome locus. *Scientific Reports*.

