# Supplementary material for: Noncoding RNA Transcripts during Differentiation of Induced Pluripotent Stem Cells into Hepatocytes
Source: Stem Cells Int. 2018 Aug 19;2018:5692840. doi: 10.1155/2018/5692840 (PMC6120260; doi:10.1155/2018/5692840)
Supplement: Supplementary Materials — Figure 1: statistics of RNASeq. (A) Total number of sequenced reads, reads after clipping, and mapped reads; both symbolised all reads obtained after sequencing. The bar showing clipped reads contains reads that could be processed by cutting the helper sequences. Mapped read bar shows reads aligned to a reference genome. Clipped and mapped reads are divided into reads containing an adapter (length below 50 nt) and reads that do not contain an adapter (length ≥ 50 nt). (B) Percentage of mapped reads with an adapter; mapped reads of miRNA and snoRNA are divided into reads containing an adapter (length below 50 nt) and reads that do not contain an adapter (length ≥ 50 nt); HLCd20 and HLCd24 show reads from HLC day 20 and HLC day 24, respectively, of differentiation, in comparison to reads from hepatocytes. Figure 2: percentage of different transcript types in the sequencing; identified ncRNA without miRNA and snoRNA transcripts which were successfully mapped and overlapped genome annotations. HLCd20 and HLCd24 show transcripts from HLC day 20 and HLC day 24, respectively, of differentiation, in comparison to transcripts from hepatocytes. Table 1: list of differentially expressed miRNA and snoRNA. Table 2: conservation of snoRNA candidates. [file 5692840.f1.zip › Supplementary Materials (fig1, fig2, table 2).docx]

Supplementary Material

Fig.1

Fig.1 Statistics of RNASeq; A: Total number of sequenced reads, reads after clipping and mapped reads; both- symbolised all reads obtained after sequencing; The bar showing clipped reads contains reads that could be processed by cutting the helper sequences. Mapped reads bar show reads aligned to a reference genome. Clipped and mapped reads are divided into reads containing an adapter (length below 50 nt) and reads that do not contain an adapter (length >=50 nt); B: Percentage of mapped reads with an adapter; mapped reads of miRNA and snoRNA are divided into reads containing an adapter (length below 50 nt) and reads that do not contain an adapter (length >=50 nt); HLCd20, HLCd24 shows reads from HLCs day 20 and day 24 of differentiation respectively, in comparison to reads from hepatocytes


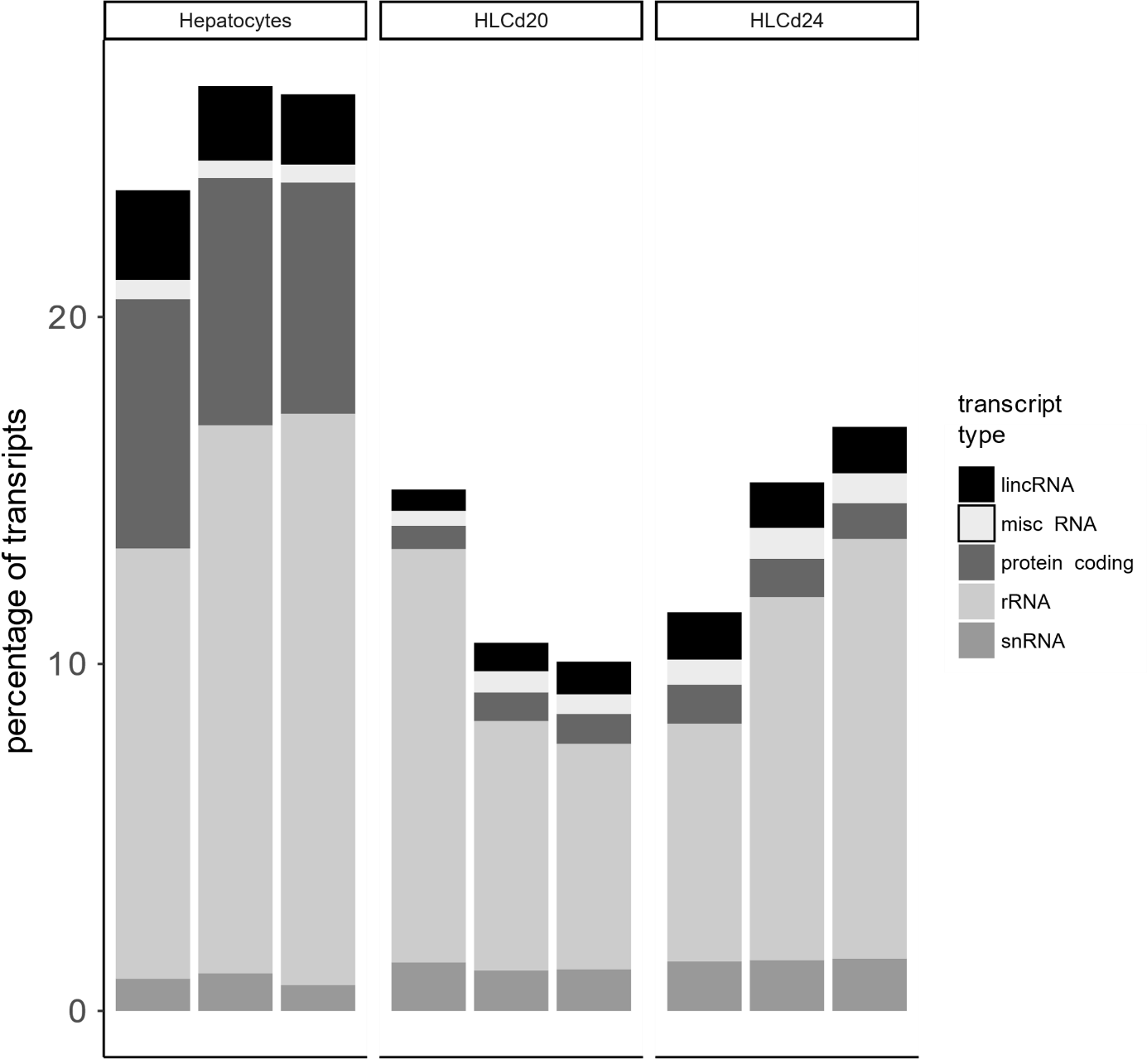


Fig.2 Percentage of different transcript types in the sequencing; identified ncRNA without miRNAs and snoRNAs transcripts which were successfully mapped and overlapped genome annotations. HLCd20, HLCd24 shows transcripts from HLCs day 20 and day 24 of differentiation respectively, in comparison to transcripts from hepatocytes

Table 1 List of differentially expressed miRNA and snoRNA

Additional file

Table 2 Conservation of snoRNA candidates

| Method | Type | Count | Conservation |
| --- | --- | --- | --- |
| snoReport | CD | 3 | Boreoeutheria |
| SnoStrip | CD | 3  1  1  1 | Primates  Human  Homininae  Boreoeutheria |
| SnoStrip | HACA | 2  1  3  2  2 | Theria  Eutheria  Primates  Boreoeutheria  Euarchontoglires |
| RNAz | - | 2  1  1 | Primates  Eutheria  Boreoeutheria |
